# Sea-level rise induced amplification of coastal protection design heights

**DOI:** 10.1038/srep40171

**Published:** 2017-01-06

**Authors:** Arne Arns, Sönke Dangendorf, Jürgen Jensen, Stefan Talke, Jens Bender, Charitha Pattiaratchi

**Affiliations:** 1 Research Institute for Water and Environment, University of Siegen, Paul-Bonatz-Str. 9-11, 57076, Siegen, Germany; 2 Civil and Environmental Engineering Department, Portland State University, 1930 SW Fourth Avenue, Portland, OR, 97201, USA; 3 School of Civil, Environmental and Mining Engineering & The UWA Oceans Institute, The University of Western Australia, 35 Stirling Highway, Crawley 6009, Australia

## Abstract

Coastal protection design heights typically consider the superimposed effects of tides, surges, waves, and relative sea-level rise (SLR), neglecting non-linear feedbacks between these forcing factors. Here, we use hydrodynamic modelling and multivariate statistics to show that shallow coastal areas are extremely sensitive to changing non-linear interactions between individual components caused by SLR. As sea-level increases, the depth-limitation of waves relaxes, resulting in waves with larger periods, greater amplitudes, and higher run-up; moreover, depth and frictional changes affect tide, surge, and wave characteristics, altering the relative importance of other risk factors. Consequently, sea-level driven changes in wave characteristics, and to a lesser extent, tides, amplify the resulting design heights by an average of 48–56%, relative to design changes caused by SLR alone. Since many of the world’s most vulnerable coastlines are impacted by depth-limited waves, our results suggest that the overall influence of SLR may be greatly underestimated in many regions.

Coastal flood risk and erosion is exacerbated by SLR, changes in storminess and other climatic effects and can be further accentuated by anthropogenic interventions^1^,^2^,^3^ such as channel deepening, wetland reclamation, and harbor development. Designing sufficient coastal protection structures that account for SLR and prevent frequent inundation is thus an essential component of modern and resilient coastal societies.

Coastal protection measures are typically designed to withstand storm tides (i.e. total still water levels caused by astronomical tides and wind-induced surge excluding wave setup), wave-run-up, and the pounding from waves. Local average recurrence intervals (ARI) of both storm tides and waves (often modified by consideration of site specific wave run-up height, i.e. the vertical excursion of a design wave on a structure) are usually calculated independently using some form of statistical assessment^4^ and then recombined to produce a design height that protects against extreme events of certain probability, as e.g. the one in 100 year ARI (hereafter ARI_100_). Such assessment procedures provide adequate protection under present-day conditions, but fail to account for future climate change^5^,^6^,^7^. A simple but commonly used approach to account for climate effects is to increase the height of coastal defenses by an amount equivalent to the regionally projected SLR^8^. Since trends in past and future storminess (meteorological forcing) are still uncertain and remain difficult to separate from natural variability on a local scale^9^, the pragmatic SLR-only approach may therefore be appropriate in many cases. However, we demonstrate that coastal regions bounded by shallow continental shelf areas (i.e. areas with mild slopes or extensive tidal flats) are sensitive to a number of common non-linear feedbacks induced by SLR. These non-linear effects can alter wave heights, tide characteristics, and surge magnitudes, and must be considered in risk assessments to maintain the integrity of coastal defenses^10^,^11^.

In some regions around the world, tidal ranges (the difference between high and low water tide levels) are increasing over time due to SLR-induced depth changes, which alters frictional damping and other shallow water effects^11^,^12^,^13^,^14^. By contrast, storm surge (i.e. the non-tidal residuals) can both amplify^3^ or diminish^13^ with SLR due to the decreased effect of bottom friction^3^ or surface wind stress^13^ on the water column. Surface gravity waves (period <20 seconds) become amplified: as sea-level increases, waves that previously were breaking in deeper water impinge on coastal defenses, increasing the wave run-up height^15^. Here, we investigate such non-linear effects of SLR on coastal design heights exemplarily in the shallow coastal areas of the German Wadden Sea that is located in the southeastern North Sea (Fig. 1a,b).

Nonlinear effects are assessed using a storm tide model which combines the coupled effects of tides, surge, and wind waves with an average horizontal resolution of 1 km. The model is forced with the 20^th^ Century Reanalysis meteorological data^16^ and local SLR^17^ from 1970–2013, and was previously validated against observational data^13^ (see also Fig. 1s). We next apply the same meteorological forcing to three SLR scenarios: the median of estimates by 2100 associated with RCP4.5, RCP8.5^18^ and a high-end scenario based on RCP8.5^19^ (hereafter RCP8.5HE) (see Methods) (Fig. 1c). Coastal design heights are estimated from common run-up formulas (see ref. 20) using storm tides and waves as input (see Methods). All relevant storm tide and wave combinations at the ARI_100_ are obtained introducing a novel application of bivariate design height assessment based on copulas (e.g. ref. 21), considering that storm tide and wave magnitudes are partially correlated (see Methods). As shown in Fig. 2a, the multivariate copula analysis yields an ensemble of events, each instance of which describes a particular design height resulting from combinations of storm tides and waves that together have a 0.01 annual probability of occurrence (i.e. the ARI_100_). From the “elbow shaped” contour line, we choose the most-damaging ARI_100_ event, i.e. the one causing the largest wave run-up height. These coastal design heights (red circles in Fig. 2a) are the most-likely source of dike-failure^20^.

## Results

### Water level changes

**Figure 1:**
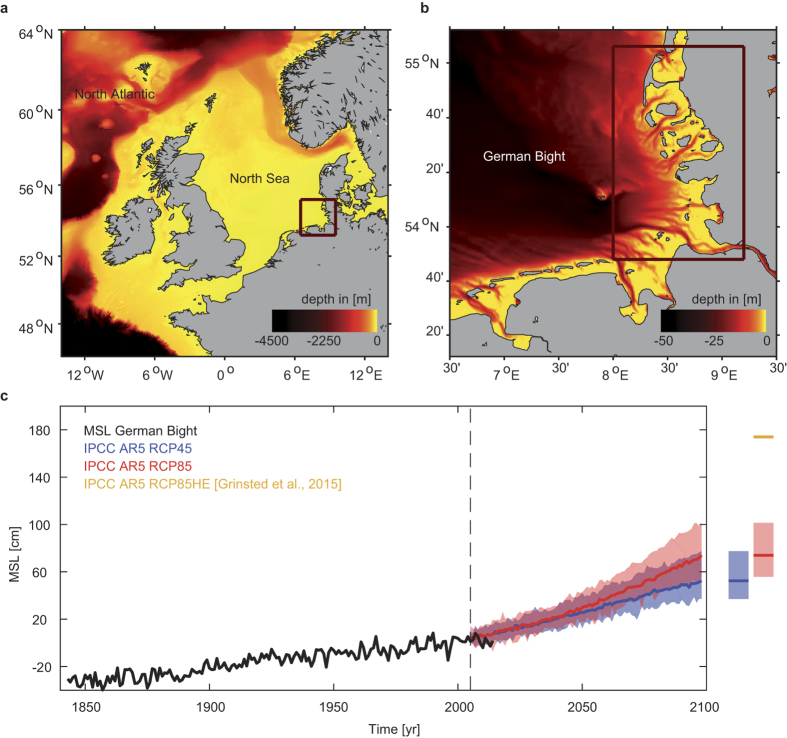
Study area and SLR projections. (**a**) Geographical boundaries of the numerical model used to simulate storm tides and waves in the main study region. Our assessment specifically focuses on the shallow shelf areas of the German Wadden Sea, which is highlighted in (**b**). Colour contours in (**a**) and (**b**) represent the bathymetry. (**c**) Shown are observed (black) and modelled (coloured) sea-level changes from ref. 18 following RCP4.5, RCP8.5 and RCP8.5HE scenarios. The maps (**a**) and (**b**) are generated using MATLAB 2015b (http://mathworks.com).

**Figure 2:**
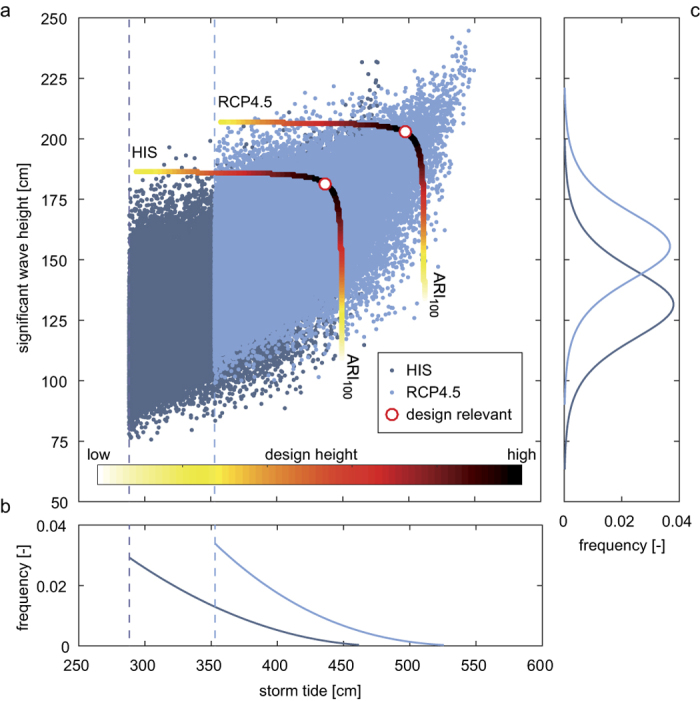
Coastal design heights of the ARI_100_ from a bivariate copula assessment. (**a**) Shown are 10,000 random samples of storm tides and wave heights at western Pellworm considering both present day (dark blue dots) and possible future (here RCP4.5) conditions (light blue dots). The colour bar describes different design heights marked on the ARI_100_ contour lines resulting from different bivariate samples. The red circle indicates the design relevant combination, i.e. the largest total design height. (**b**) Marginal distributions (GPD) of the univariate samples of storm tides. (**c**) As (**b**) but for wave heights (Gaussian).

**Figure 3:**
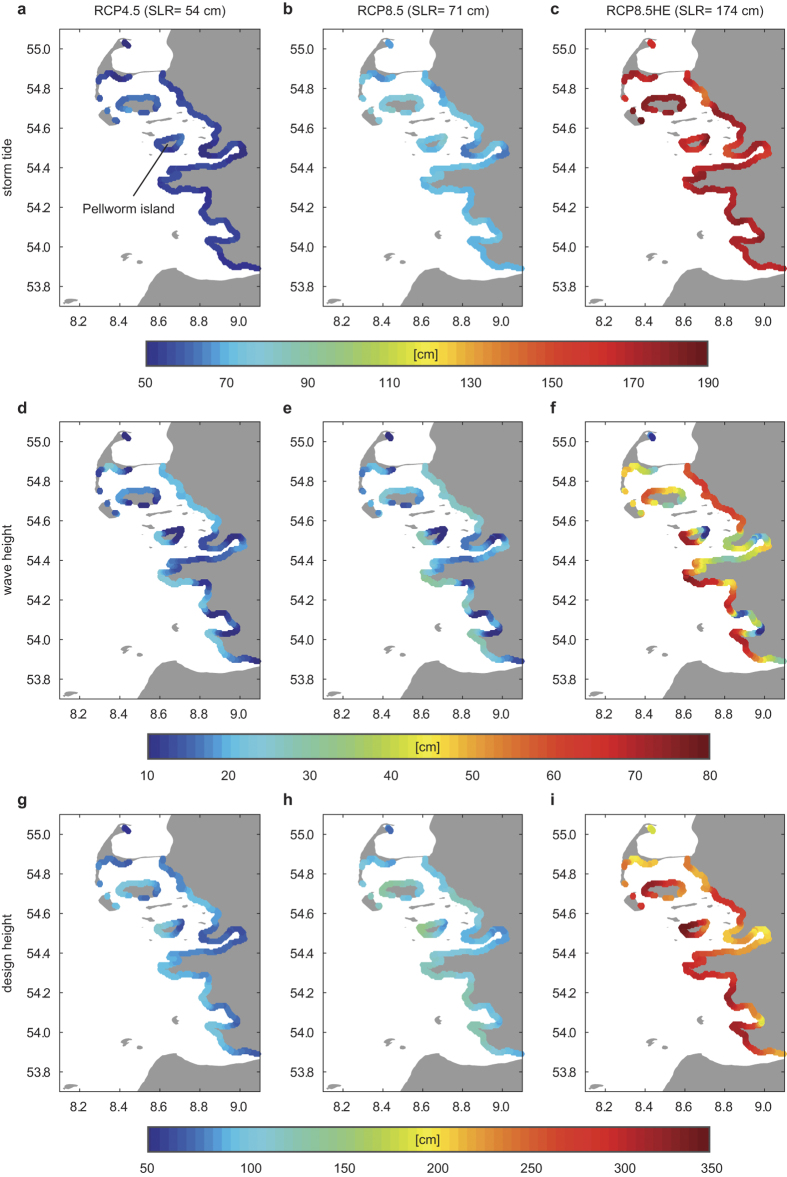
ARI_100_ changes in storm tides, waves, and design heights due to SLR. Shown are absolute changes in the ARI_100_ compared to historical (1970–2013) conditions (HIS) under RCP4.5 (left column), RCP8.5 (middle column), and RCP8.5HE (right column) for storm tides (**a–c**), wave heights (**d–f**), and design heights (**g–i**). Note the different ranges in the colorbars. The maps are generated using MATLAB 2015b (http://mathworks.com).

Our simulations show that storm tide levels outpace SLR at most locations (Fig. 3a–c). Under RCP4.5 (Fig. 1c), storm tides at the ARI_100_ increase by up to 0.67 m, exceeding the expected SLR (0.54 m) by up to 0.13 m. Similar excess values of 0.14 and 0.17 m are observed under RCP8.5 (SLR = 0.71 m) and RCP8.5HE (SLR = 1.74 m) scenarios, respectively. The changes are spatially heterogeneous and non-linear increases tend to be the least in areas adjacent to large tidal streams, i.e. where depths are larger (see Figs 1b and 3a–c). Changes in storm tides can be explained by a reduction of both the effective friction and shallow water effects. In linearized tidal equations, the friction term is inversely proportional to depth (e.g. ref. 20). Assuming that sea-level increases without geomorphic adjustment (our assumption), shallow water areas will increasingly be subject to less frictional damping and tidal deformation^13^. Wave-current interaction (at the surface and bed) changes as the water level increases, also altering energy dissipation in the sea. This decreases the observed damping and depth dependence of tides during storm events, and helps explain why tidal ranges increase (Fig. 2s) (see also ref. 13).

The increase in tidal range counteracts the meteorological component of the storm tide (i.e., surge), which is observed to decrease by 1.8% (RCP4.5), 2.3% (RCP8.5), and 5.1% (RCP8.5HE) on average (not shown here) as sea-level rises. Storm surge is subject to the same constraints as tides, and reduced bottom friction or increased depth can amplify the surge in estuaries (e.g. ref. 3); in the North Sea, however, the decreased bottom friction appears to be counteracted by the lessened effectiveness of surface wind stress. Basically, the same wind forcing (surface stress) is less effective at dragging water and produces a smaller surge when water becomes deeper.

**Figure 4:**
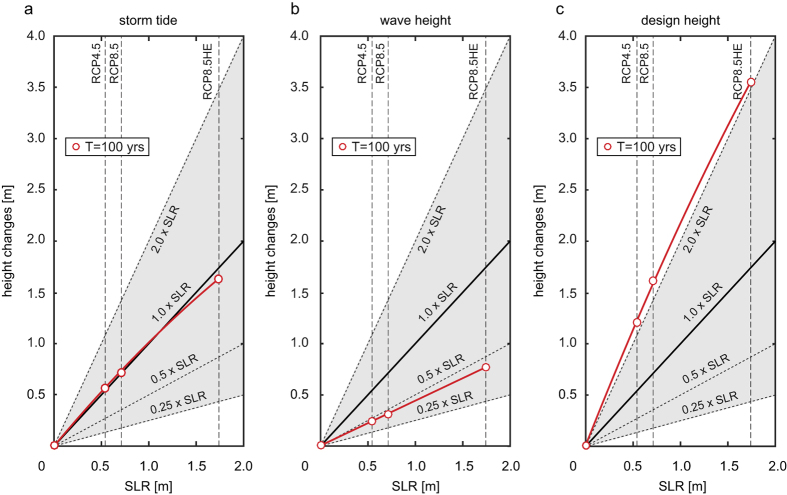
Relationship between different SLR projections and storm tides, waves, and design heights at western Pellworm. For all RCP based SLR projections the feedback in the ARI_100_ (red lines) of (**a**) storm tides; (**b**) wave heights; and, (**c**) design heights is shown. Dashed lines mark changes compared to SLR covering scaling factors equal to 0.25, 0.5, 1.0 and 2.0.

Overall, tidal changes outweigh surge changes (Fig. 4a). This effect is illustrated on the western side of the Wadden Sea island Pellworm, where the mean tide range over all considered storm events (mean storm tide range) increases by 0.32 m (RCP4.5), 0.4 m (RCP8.5), and 0.77 m (RCP8.5HE) (Fig. 2s), a phenomenon consistent with observational evidence in this region (e.g. refs 14 and 22). Although the amplification is more pronounced in the tidal low water levels (Fig. 2s), the tidal high water levels (which are relevant to design) of almost all scenarios significantly increase by a factor of up to 1.24 faster than when SLR is considered alone (see Fig. 4a and Table 1s). Interestingly, the predicted sensitivity of tides to an incremental increase in sea-level diminishes when depths become large; as a result, in the extreme RCP8.5HE scenario, depth-induced decreases in surge outweigh increases in high water. These observations again highlight the complexity on non-linear interactions.

### Wave changes

Simulations suggest that waves and wave run-up height are much more sensitive to SLR than tides or surge (Fig. 3d–f), for the shallow bathymetry considered here. At exposed, westward oriented locations, wave magnitudes at the ARI_100_ exceed modern conditions by up to 0.25 (RCP4.5), 0.33 (RCP8.5), and 0.78 m (RCP8.5HE) (Fig. 3d–f). Positive increases are observed even in the more protected areas on the lee-side of barrier Islands.

**Figure 5:**
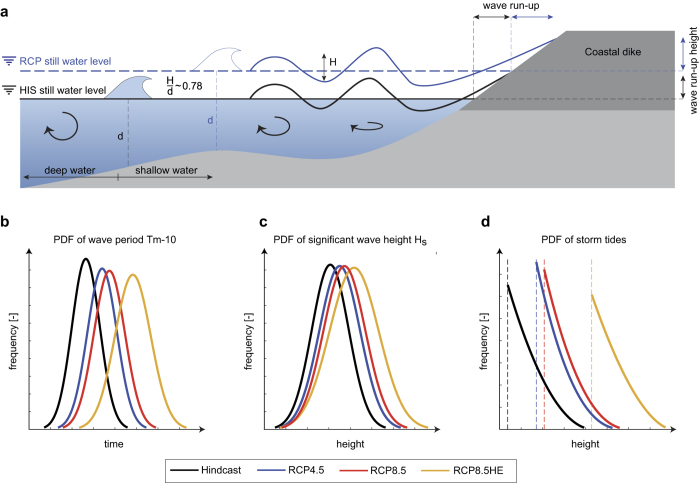
Schematic of depth related changes in wave propagation towards the coast due to SLR. (**a**) Waves travelling in the open ocean typically cover a range of heights. In flat water, individual wave breaking is inevitable if the water depth becomes too shallow. Under HIS conditions (black line), wave breaking occurs further off the coastline. Remaining waves are affected by depth limitations allowing only those waves to travel to the coastline being less than ~0.78 times the water depth (simplified assumption, here only used for explanation purposes, see e.g. ref. 23). Under RCP conditions, fewer waves are affected by wave breaking facilitating access to the coast. Probability density functions (PDF) of (**b**) wave periods (Gaussian); (**c**) wave heights (Gaussian); and (**d**) storm tides (GPD) at western Pellworm describing the relative likelihood of each component to take on a given value. In (**b,c**), PDF changes from HIS to RCP based SLR projections are highlighted indicating changes in the location parameter and the variance of each component.

The observed amplification in wave run-up is driven by a predicted decrease in wave breaking away from the coast (see Fig. 5a). Depth-induced breaking occurs when waves propagate into very shallow areas, and the wave height can no longer be supported by the water depth. An empirically based criterion states that wave breaking occurs at an average breaker parameter γ (wave height to depth ratio) of ~0.78 (here only used to explain general mechanisms), but significant variation is observed with wave conditions and the bathymetry^23^. As sea-level increases, fewer waves exceed this criterion. Hence, for a given wave period, larger waves impinge on coastal defenses, to a degree related to the percent SLR. Moreover, increased depth allows longer period waves to reach the coast, due to decreased non-linearity and wave steepness at low frequencies. Both effects increase the amount of energy impinging on coastal defenses, and produce a shift in both the significant wave height and its period (see Fig. 5b,c), each causing an increase in run-up height (see Methods). Effectively, as Fig. 5a schematically shows, sand-flats and shallow sub-tidal areas currently act as a high-pass filter of the wave-climate existing on the open sea, allowing only relatively weaker, smaller period waves to the coast (see e.g. ref. 20). The magnitudes of the waves are modulated by the phase of the tidal cycle within each simulated storm event. Waves at the coast are smallest during low water due to the filtering effect, remain low during ebb and flood due to wave-current interaction effects, but are elevated during low current, high water periods (Fig. 3s). As SLR continues, the natural bathymetric protection caused by shallow water reduces and coastal structures will increasingly observe more open-ocean like wave conditions. This effect is also evident in our modeled dependency between storm tides and waves; typically, a larger storm tide results in larger wave magnitudes, up to a ‘saturation’ depth at which waves are no-longer filtered. As sea-level increases, the same saturation depth is reached for smaller storm tides. Therefore, the observed Kendall’s rank correlation coefficient between waves and storm-tides decreases from 0.4 (HIS) to 0.37 (RCP4.5), 0.36 (RCP8.5), and 0.32 (RCP8.5HE). In effect, waves and storm tides become more independent with SLR, subtly affecting the bivariate statistics.

### Design height changes

The joint, coupled influence of changing storm tides and waves on design heights is highlighted in Fig. 3g–i. Along exposed westward oriented coastlines, changes in design heights clearly exceed the simultaneous SLR, with values ranging from 56 to 122 cm (RCP4.5), 73 to 157 (RCP8.5), and 181 to 357 cm (RCP8.5HE). Over the entire region, predicted changes in design heights exceed SLR by an average of 48–56% (slightly decreasing with larger SLR scenarios) (Fig. 4s and Table 1s). At exposed locations such as Pellworm Island, the change in design height is more than doubled relative to SLR alone (Fig. 4c, Fig. 4s, and Table 1s).

## Discussion

Our results have broad implications for coastal impact studies: until recently, climate change studies have largely focused on the effects of SLR^8^,^18^ and/or changing storm tracks^7^,^9^. However, our analysis shows that in shallow coastal areas, wave heights, tides, and surges are strongly correlated with SLR, and with each other. To obtain design heights and assess risk, these non-linearities need to be considered by coupled, multivariate assessment such as the copula approach used here. Since many of the densely populated and highly vulnerable world’s open coasts are fringed by shallow shelfs, present-day coastal bathymetry likely provides significant protection against wave attack. If SLR is not accompanied by morphodynamic adjustment (e.g. increased deposition), the feedback effects highlighted here are likely to occur, and coastlines worldwide will be more subject to open-ocean conditions. An example is the US Gulf Coast around Louisiana and Texas, where loss of wetlands and intertidal areas is having a significant impact on surge risk^24^. Simple allowances from uncertain SLR projections^8^, as adopted by the Fifth Assessment Report of the IPCC^5^, therefore tend to underestimate the impact of future SLR on the required coastal design heights at many locations around the world.

The investigations are based on a number of assumptions introducing uncertainties in the potential feedback of design heights to SLR. This includes but is not limited to the wave run-up formula we used (see e.g. ref. 25), the assumption that coastal bathymetries and coastlines do not change with SLR (see e.g. refs 26 and 27), or the uncertainties in future SLR which directly affect future coastal extremes^28^. Furthermore, the results presented here are for the specific case study of an impermeable 1:6 slope dike and similar assessments need to be adapted to other locations.

However, the general processes identified here—changing storm tides and wave heights—are endemic worldwide. Many locations such as the South China Sea or the Arctic are, as the German North Sea Coast, relatively shallow, semi-enclosed ocean basins that are subject to significant waves and altered tides. To plan for the future and protect vital coastal areas, current best-practice needs to be revised to include the effects of non-linear, but coupled changes in risk. The effects, as shown by the large spatial variability observed in Fig. 3, depend on the complex effects of the bathymetry on waves, surges, and tides, and may be further influenced by changes in the morphodynamic equilibrium condition and anthropogenic interventions (e.g., beach nourishment). Nonetheless, the precautionary principle suggests that the approach used here –numerical modeling and multivariate analysis under the assumption of constant depth changes everywhere– will provide significantly better design heights and protection than current methodologies.

## Methods

### Numerical model set-up

Storm tides, tides, and waves are simulated using MIKE 21 FM HD, a coupled, depth averaged, hydrodynamic/wave model developed by the Danish Hydraulic Institute (see ref. 13). The model domain covers the entire North Sea and part of the adjacent North Atlantic and accounts for large scale meteorological and hydrodynamic effects^13^. In the coastal region, seabed topography at ~15 m resolution was obtained from the *Schleswig-Holstein Agency for Coastal Defense, National Parks and Marine Conservation* (LKN-SH). In the remaining domain, bathymetry at 30 arc-second intervals was obtained from the *General Bathymetric Chart of the Oceans* (GEBCO), which is produced by the *British Oceanographic Data Centre* (BODC). At the open boundary the model is forced with astronomical tides varying in time at a total number of 127 points along the domain. Tide levels have been calculated using the MIKE internal tide model. MSL rise at the boundary is forced using historical observations^17^ and RCP projections^18^,^19^ for present-day and future conditions by 2100 added on to the observed SLR, respectively. Waves and storm tides for all scenarios are produced by applying continuous wind and pressure fields from reanalysis data for the years 1970–2013^16^. This time period was chosen due to the availability of high quality *in-situ* data but also to include the 1976 storm tide, the largest event ever recorded in many places of the German Bight. Model runs were output every 10 minutes. For every scenario, the 99.7^th^ percentile water level exceedances are then estimated at ~500 m increments along the ~470 km North Sea coastline in Schleswig Holstein.

### SLR projection

We consider the median of three different SLR projections by 2100 associated with the RCP4.5^18^, RCP8.5^18^, and RCP8.5HE^19^ (high end). All projections represent the effects of thermosteric and halosteric density changes, the response of the ocean to wind and pressure forcing, changes in ocean mass (Greenland and Antarctic ice sheets, glaciers, and groundwater), and glacial isostatic adjustment (see the Fifth Assessment Report of the IPCC^8^ for details). In addition, the RCP8.5HE projection includes rapid ice melt in the Antarctic, a plausible but more extreme sea level rise scenario^29^ that should nonetheless be considered from a coastal decision-making and management point of view. At a central point in the German Bight, these projections suggest a mean SLR of 0.54 m, 0.71 m, and 1.74 m by 2100 under RCP4.5, RCP8.5, and RCP8.5HE, respectively.

### Extreme value statistics

Extreme value statistics are used to infer magnitudes of both storm tides and waves at specific ARI’s. We employ the Peak Over Threshold (POT) method^4^,^30^ and fit the following generalized Pareto distribution (GPD) to a ranked list of independent events exceeding a specified threshold of simulated high water peaks,


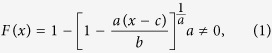



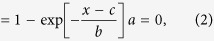


where *c* is the location (threshold) parameter, *b* is the scale parameter, *a* is the shape parameter and the threshold of exceedances is *x*. The parameters are estimated using the Maximum Likelihood Estimation (MLE) method^4^, with the threshold level of 99.7^th^ percentile yielding consistent and stable results in the German Bight^30^. A declustering scheme based on the extremal index^4^ ensured that data were independent. Wave heights are described using the GPD but also a range of other common distribution functions including the Lognormal, Normal (Gaussian), Exponential, Weibull, and Generalized Extreme Value (GEV) distribution (see e.g. ref. 4). The best fitting distribution is assessed by calculating the minimum RMSE between the theoretical and empirical wave distributions.

The two univariate marginal distributions of storm tides and waves where then applied to assess their joint magnitudes and frequencies. We used Archimedean Copulas to describe the dependence between the two marginal distributions^31^, and hence the bivariate ARI’s. Specifically, for each SLR scenario and coastal grid point, we first obtain coincident samples of peak storm tides and wave heights in a window that is ± 120 minutes from the predicted high tide (Fig. 4s). The marginal distributions for storm tides (Fig. 2b) and waves (Fig. 2c) are obtained using univariate analysis. The dependence of storm tides and waves in our modelled data sets are then assessed using Kendall’s rank correlation. The correlation coefficient then becomes an input parameter in our copula analysis. Next, three types of copulas (Gumbel-, Clayton-, and Frank Copula) are evaluated and the model with the minimum RMSE between the parametric and the empirical copula^32^ is retained to estimate bivariate ARI’s. To qualitatively assess whether results are reasonable, 10,000 random events are generated from the parametric copula and the marginal distributions and compared to the numerical model data for consistency (see e.g. ref. 21 for an example).

Next, bivariate contour lines for the ARI_100_ are calculated, resulting in a family of possible combinations which have the same recurrence interval. For example, in Fig. 2a, a small storm tide (300 cm) with large waves (185 cm) has the same historical ARI as a large storm tide (450 cm) with small waves (~100 cm; see HIS case). Wave run-up height (see next section) is then calculated for each ARI_100_ event (elbow shaped contour line in Fig. 2a). The maximum overall height (i.e. storm tide plus wave run-up height) is assumed to be design relevant (see the red dot in Fig. 2a) and differences between the HIS and scenario runs indicate SLR induced changes in design heights.

### Run-up

Dikes are constructed to withstand the impact of extreme water levels and waves. Potential dike failures result from several mechanisms, including overflow induced by elevated water levels and dike breaching caused by wave overtopping. In practice, dikes are built to withstand the wave run-up height *R*_*u,2%*_, the vertically measured distance which is exceeded by 2% of all incident waves^20^. Along the German North Sea coast, we assume all dikes consist of smooth embankments with a 1:6 slope (see Fig. 5a), following current recommended best practice. However, in reality the dike slopes slightly vary along different coastal stretches, and would need to be considered in a site-specific assessment.

Though extensively studied, uncertainty and bias is still found when wave run-up formulas are compared to physical test results (see e.g. ref. 20; and references therein). Here, we focus on relative wave run-up height changes from different SLR scenarios to minimize the effect of these uncertainties. Our assessment is based on a formula provided in ref. 20 describing the wave run-up on smooth and straight slopes (assuming all waves to attack perpendicular and in relatively deep water at the dike toe but without any wave breaking in front of the dike^20^), where the relative wave run-up height is calculated as


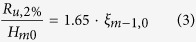


where H_m0_ is the wave height at the toe of the structure. The breaker parameter ξ_m−1,0_ is defined as


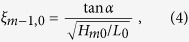


where α is the outer dike slope and the deep water wave length L_0_ is given by


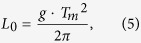


where g is the gravitational acceleration and the modal wave period T_m_ as obtained from the scenario and HIS runs.

## Additional Information

**How to cite this article**: Arns, A. *et al*. Sea-level rise induced amplification of coastal protection design heights. *Sci. Rep.* **7**, 40171; doi: 10.1038/srep40171 (2017).

**Publisher's note:** Springer Nature remains neutral with regard to jurisdictional claims in published maps and institutional affiliations.

## Supplementary Material

Supplementary Information
