# Supplementary material for: Sea-level rise induced amplification of coastal protection design heights
Source: Sci Rep. 2017 Jan 6;7:40171. doi: 10.1038/srep40171 (PMC5216410; doi:10.1038/srep40171)
Supplement: Supplementary Information [file srep40171-s1.pdf]

Sea-level rise induced amplification of coastal protection design heights

Supplementary information

Arne Arns[1], Sönke Dangendorf[1], Jürgen Jensen[1], Stefan Talke[2], Jens Bender[1], and Charitha Pattiaratchi[3]

[1]Research Institute for Water and Environment, University of Siegen, Paul-Bonatz-Str. 9-11, 57076, Siegen, Germany

[2]Civil and Environmental Engineering Department, Portland State University, 1930 SW Fourth Avenue, Portland, OR, 97201, USA

[3]School of Civil, Environmental and Mining Engineering & The UWA Oceans Institute, The University of Western Australia, 35 Stirling Highway, Crawley 6009, Australia

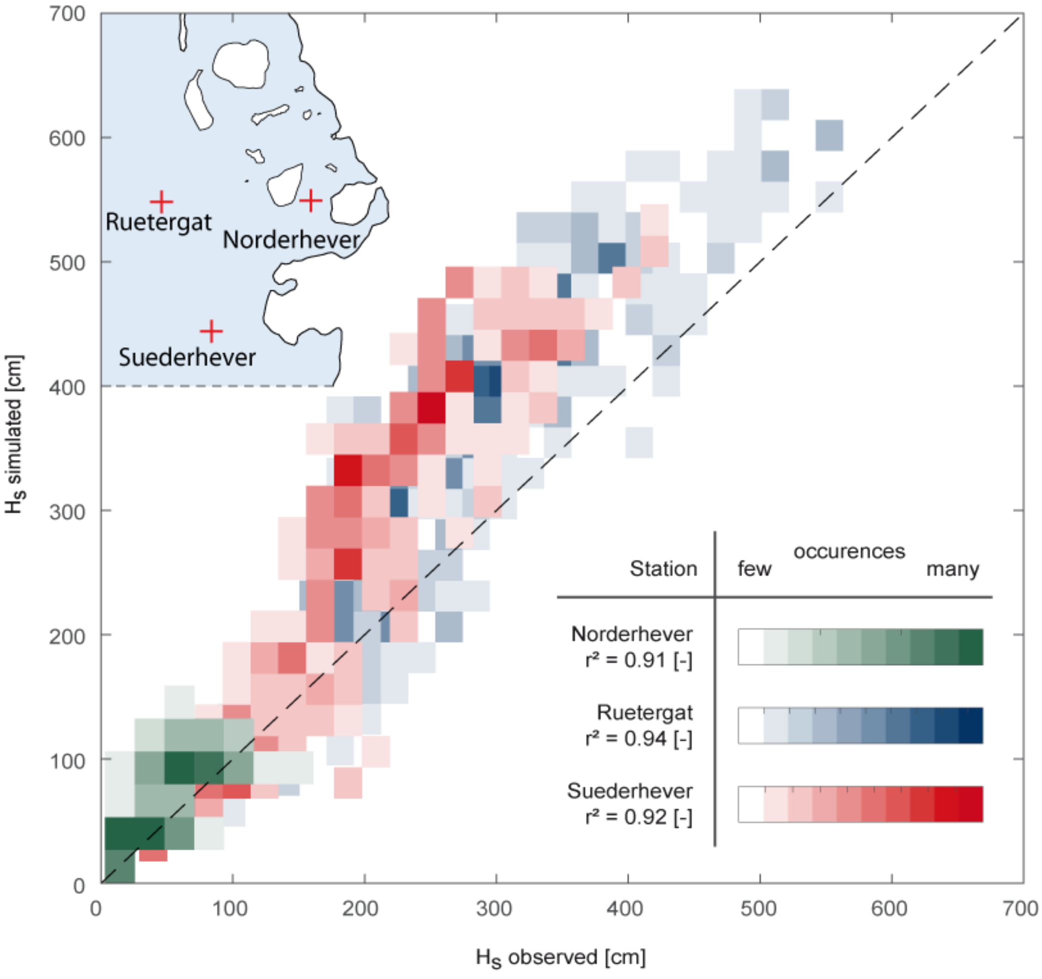

Fig. 1s | Simulated vs. observed significant wave heights. Comparison between observed and simulated significant wave heights at the wave buoy stations Norderhever (green), Rutergat (blue), and Suederhever (red). The locations of the wave buoys are shown in the upper left part of the figure. The models performance in terms of r^2 (i.e. the coefficient of determination) at all three station is given in the legend. The map is generated using MATLAB 2015b (http://mathworks.com).

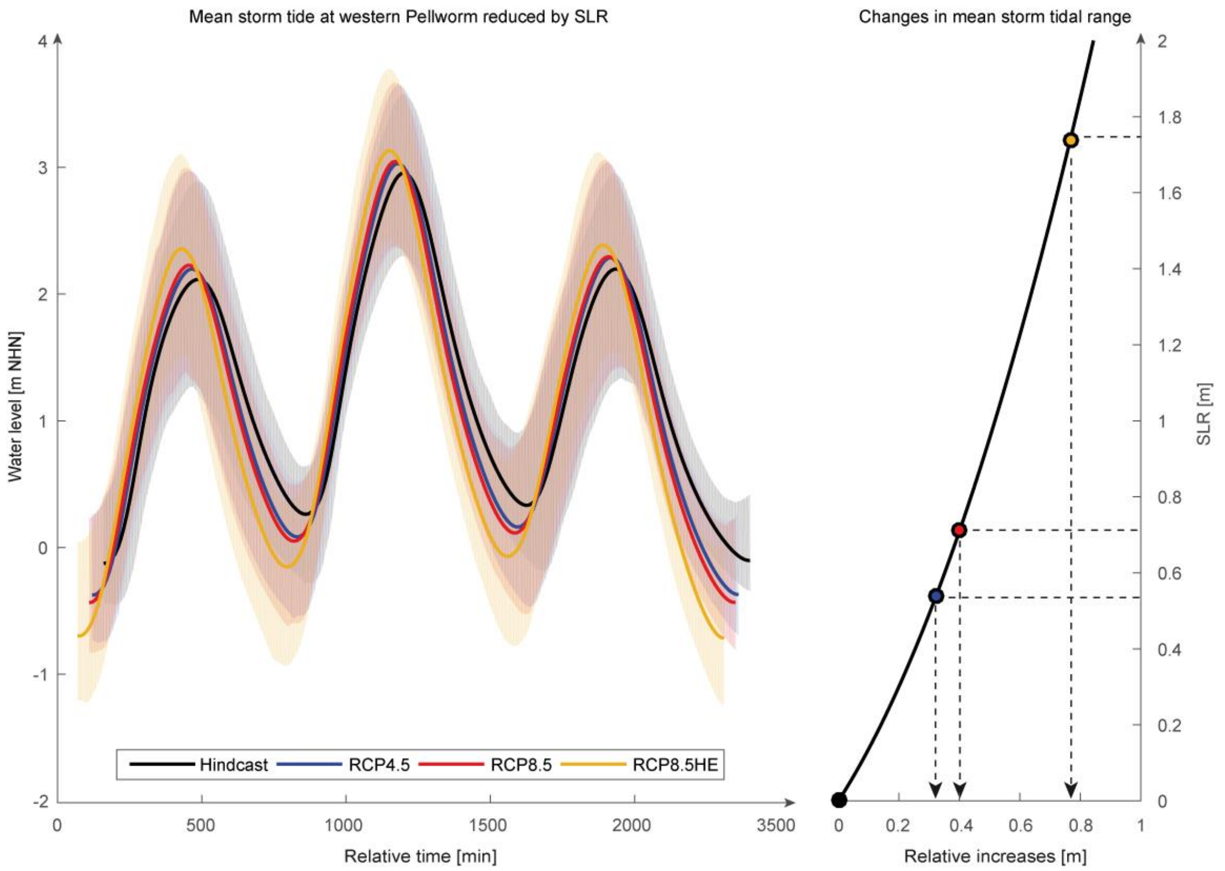

Fig. 2s | Mean tide changes under different scenarios. Mean modelled behaviour of the tide wave cycle before, during, and after the peak of the storm tide at the gauge in western Pellworm Island. Black curves indicate the hindcast (1970-2013) situation while red/blue/yellow refer to the RCP 4.5/8.5/8.5HE scenarios, respectively. The curves indicate a SLR induced growth in tidal oscillations that is observed in both mean high water and mean low water. Consequently, the tidal range increases by 0.32 to 0.77m (x-axis) for different RCP scenarios (y-axis; plot at right). The envelopes from all 75 events (i.e. the range of possible values) at given scenarios are highlighted as colour shaded areas (left plot).

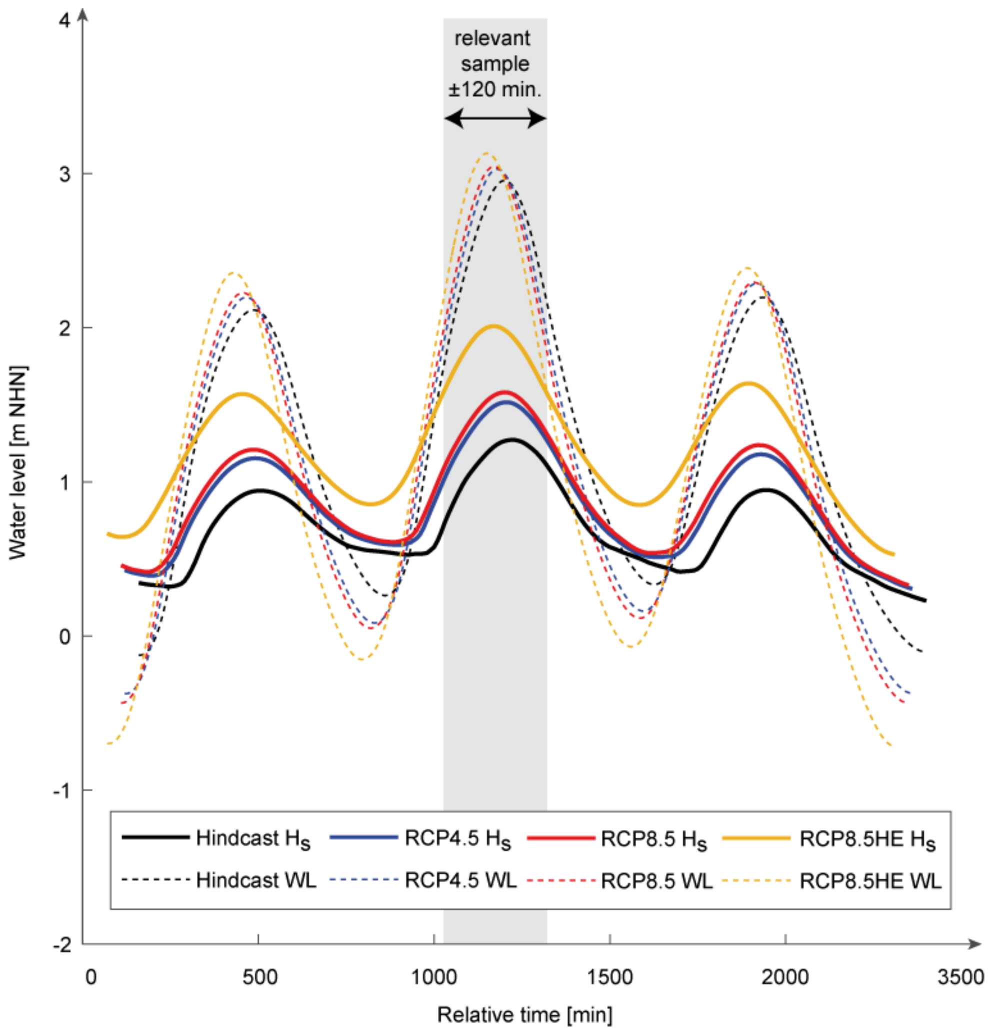

Fig. 3s | Waves vs. mean tide changes. Modelled significant wave heights (solid lines) vary over the tidal cycle (reduced by SLR; dashed lines, see also **Fig.2s**) at western Pellworm. Colours denote RCP scenarios (see **Fig. 2s**). The figure highlights the depth dependency of shallow water waves. As sea-level rises, the waves' depth dependency decreases and results in larger waves throughout the tidal cycle. The grey shaded area describes the sampling method used to create our uni- and multivariate extreme value statistics. For each event, we consider the largest significant wave height which occurred within ±120 minutes from the storm tidal high water.

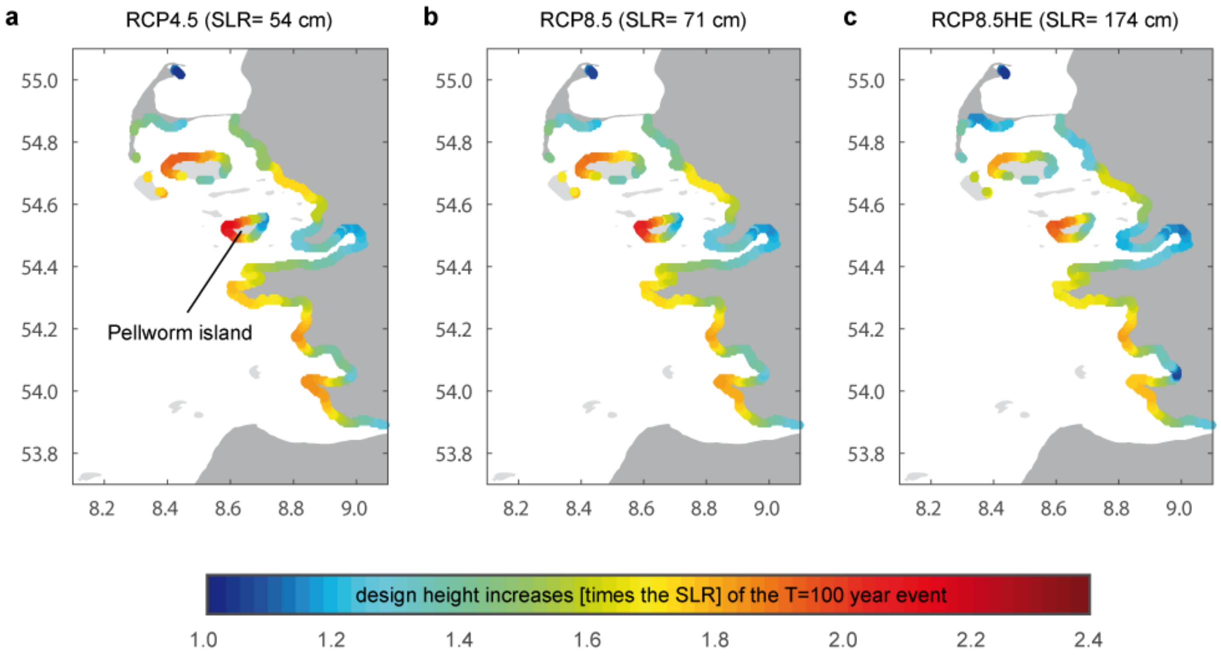

Fig. 4s | Factorial design height increases under different SLR projections. Figures (**a-b**) highlight design height increases at the ARI_{100} as relative factor compared to SLR under different RCP's. Over all RCP's, the average factorial increase amounts to 48-56% (slightly decreasing with larger SLR projections) but in exposed locations, such as Pellworm Island, the change in design heights is more than doubled relative to SLR alone. The maps are generated using MATLAB 2015b (http://mathworks.com).

Table 1s | Factorial changes of storm tides, wave heights, and design heights. Storm tide, wave height, and design height increases as a relative factor compared to SLR under different RCPs, calculated for ARI's of 50, 100, and 200 years at western Pellworm.

location		western Pellworm			study area mean			study area maximum		
ARI's		50-yrs	100-yrs	200-yrs	50-yrs	100-yrs	200-yrs	50-yrs	100-yrs	200-yrs
storm tide	RCP4.5	1.04	1.04	1.04	1.04	1.03	1.01	1.23	1.24	1.33
	RCP8.5	1.03	1.01	1.00	1.03	1.01	1.00	1.21	1.20	1.22
	RCP8.5HE	1.00	0.96	0.91	1.01	0.99	0.96	1.11	1.10	1.10
wave height	RCP4.5	0.46	0.46	0.45	0.28	0.29	0.29	0.47	0.47	0.50
	RCP8.5	0.44	0.44	0.44	0.27	0.27	0.28	0.45	0.46	0.46
	RCP8.5HE	0.44	0.44	0.44	0.26	0.27	0.28	0.44	0.45	0.45
design height	RCP4.5	2.22	2.25	2.28	1.56	1.56	1.55	2.22	2.25	2.28
	RCP8.5	2.18	2.20	2.22	1.54	1.53	1.52	2.19	2.21	2.22
	RCP8.5HE	2.01	2.04	2.01	1.49	1.48	1.45	2.23	2.05	2.02
